# The frailty, outcomes, recovery and care steps of critically ill patients (FORECAST) study: pilot study results

**DOI:** 10.1186/s40635-022-00446-7

**Published:** 2022-06-10

**Authors:** John Muscedere, Sean M. Bagshaw, Gordon Boyd, Stephanie Sibley, Patrick Norman, Andrew Day, Miranda Hunt, Darryl Rolfson

**Affiliations:** 1 Department of Critical Care Medicine, Queens University, Kingston Health Sciences Center, 76 Stuart Street, Kingston, ON K7L 2V7 Canada; 2 Department of Critical Care Medicine, University of Alberta, Edmonton, Canada; 3 Kingston Health Sciences Center, Kingston, ON Canada; 4 University of Alberta, Edmonton, Canada

**Keywords:** Frailty, Clinical frailty scale, Frailty index, Critical care outcomes, Adverse events, Care processes

## Abstract

**Introduction:**

Frailty is common in critically ill patients and is associated with increased morbidity and mortality. There remains uncertainty as to the optimal method/timing of frailty assessment and the impact of care processes and adverse events on outcomes is unknown. We conducted a pilot study to inform on the conduct, design and feasibility of a multicenter study measuring frailty longitudinally during critical illness, care processes, occurrence of adverse events, and resultant outcomes.

**Methods:**

Single-center pilot study enrolling patients over the age of 55 admitted to an Intensive Care Unit (ICU) for life-support interventions including mechanical ventilation, vasopressor therapy and/or renal replacement therapy. Frailty was measured on ICU admission and hospital discharge with the Clinical Frailty Scale (CFS), the Frailty Index (FI) and CFS at 6-month follow-up. Frailty was defined as CFS ≥ 5 and a FI ≥ 0.20. Processes of care and adverse events were measured during their ICU and hospital stay including nutritional support, mobility, nosocomial infections and delirium. ICU, hospital and 6 months were determined.

**Results:**

In 49 patients enrolled, the mean (SD) age was 68.7 ± 7.9 with a 6-month mortality of 29%. Enrollment was 1 patient/per week. Frailty was successfully measured at different time points during the patients stay/follow-up and varied by method/timing of assessment; by CFS and FI, respectively, in 17/49 (36%), 23/49 (47%) on admission, 22/33 (67%), 21/33 (63%) on hospital discharge and 11/30 (37%) had a CFS ≥ 5 at 6 months. Processes of care and adverse events were readily captured during the ICU and ward stay with the exception of ward nutritional data. ICU, hospital outcomes and follow-up outcomes were worse in those who were frail irrespective of ascertainment method. Pre-existing frailty remained static in survivors, but progressed in non-frail survivors.

**Discussion:**

In this pilot study, we demonstrate that frailty measurement in critically ill patients over the course and recovery of their illness is feasible, that processes of care and adverse events are readily captured, have developed the tools and obtained data necessary for the planning and conduct of a large multicenter trial studying the interaction between frailty and critical illness.

**Supplementary Information:**

The online version contains supplementary material available at 10.1186/s40635-022-00446-7.

## Introduction

Frailty is defined as a state of increased vulnerability resulting from reduced physiological reserve and loss of function in multiple systems reducing the ability to cope with normal or minor stressors [1]. It is associated with increased risk of physical, cognitive and functional decline, adverse health outcomes and mortality [2]. Thirty to forty percent of older individuals requiring hospitalization and treatment in Intensive Care Units (ICUs) are frail and it is associated with worse outcomes including increased hospital and long-term mortality compared to those not frail [3, 4]. As an example, the presence of frailty as measured with the Clinical Frailty Scale (CFS) has been found to be associated with increased ICU and 30-day mortality with a linear increase in mortality as the CFS increases [5]. All of the studies studying frailty in critical care populations have used the CFS, Frailty Index (FI) or the Frailty Phenotype to identify and measure frailty [6, 7].

Most of the ICU frailty studies reported to date have anchored their assessment of frailty status upon admission to ICU. The measurement of frailty during hospitalization and longitudinally after recovery from critical illness has been done in a few studies. A pilot study found that measuring frailty on hospital discharge after ICU admission with the Frailty Phenotype [8] was feasible and correlated with poor long-term outcomes. Geense et al. found that frailty measured with the CFS increased at hospital discharge as compared with admission but then declined over the follow-up period of 3 and 12 months [9]. Brummel et al. found that the majority of patients frail (CFS ≥ 5) at 3 or 12 months were not frail at ICU admission and that worsened frailty states were present in over 40% of the participants at 3 and 12 months; frailty was commonly associated high levels of disability and cognitive impairment [10]. Both of these large studies concluded that further study on associated and potential modifiable factors was required.

Although the evidentiary base linking frailty with poor outcomes is convincing, knowledge gaps exist with regard to the interaction between critical illness and frailty. First, the reasons for worse outcomes from critical illness in those who are frail remain unknown. Possible reasons include reduced homeostatic reserve, the presence of pre-existing illness or co-morbidities, premorbid loss of muscle mass or sarcopenia and pre-existing chronic inflammation associated with frailty [11, 12]. Second, it is possible that the treatment received is influenced by the presence of frailty leading to different therapies or limitation of treatment for those who are frail. Third, there is a scarcity of data on the impact of ICU treatments received on outcomes. Finally, for patients admitted to ICU without frailty who become frail from their acute illness, it is unknown if the development of frailty while critically ill has the same prognostic significance as that acquired in the community. Further, it is unknown as to how and to what degree the presence of frailty on hospital discharge predicts long-term outcomes and response to rehabilitation.

To inform these knowledge gaps, a large multicenter observational study measuring frailty at different time points, using a variety of frailty instruments, reporting on the processes of care received and long-term outcomes is required. To guide the planning of this study, we undertook a single-center pilot study and herein we report the results. We hypothesized that an adequately powered multicenter observational study measuring frailty at different time points, collecting data on the processes of care and long-term outcomes during the course of critical illness was feasible. For this pilot study we had the following objectives: (1) to evaluate feasibility based on the recruitment rate, consent rate and long-term follow-up metrics; (2) to demonstrate that frailty can be measured on hospital discharge; (3) to demonstrate that the processes of care of interest in ICU, in-hospital post ICU discharge and 6-month follow-up could be collected.

## Methods

A single-center, prospective observational pilot study of consecutive patients admitted to a tertiary care, medical-surgical ICU for treatment of life-threatening illness. We included patients over the age of 55 admitted to the ICU and receiving at least one life-support intervention for greater than 24 h. The eligible life-support interventions for this study were mechanical ventilation (invasive or non-invasive), receipt of intravenous vasopressors/inotropes or the receipt of acute dialysis greater than 24 h. We excluded patients who were in the ICU greater than 72 h; had limitations of treatment on ICU admission although an isolated no cardio-pulmonary resuscitation (CPR) order was acceptable; had a life expectancy less than 6 months; did not have family or caregivers available to collect collateral history; were not able to speak English and medical translators were not available; and had structural neurological disease necessitating the ICU admission including stroke or spinal cord pathology. In addition, we excluded patients in whom it was anticipated that 6-month follow-up would not be possible including lack of consent or inability to return for follow-up. As there were no therapeutic interventions, all care decisions were left to the treating clinicians.

We collected frailty status on ICU admission and hospital discharge using the CFS and a frailty index based on a modified geriatric assessment (Additional file 1: Appendix 1) [13, 14]. The timing of frailty assessment was anchored at 2 weeks prior to the acute illness and was defined as a CFS ≥ 5 or a frailty index ≥ 0.20. All data were collected from surrogates unless the patient was able to directly provide the data. We collected data at ICU admission including severity of illness (APACHE II [15]), co-morbidities (Charlson Co-Morbidity Index [16]), activities of daily living (Katz Index of Independence in Activities of Daily Living [17]) and the presence of cognitive dysfunction (The Informant Questionnaire on Cognitive decline in the elderly (IQCODE) [18]). During the patient’s ICU stay, we collected daily data on the treatment provided, including nutrition (protein, calories received), sedation/analgesia regimens (sedative and analgesic agents administered, mobility (ICU Mobility Scale [19]), and involvement of physiotherapy on that day of care. Further we collected daily data on the occurrence of adverse events including delirium (Confusion Assessment Method for the Intensive Care Unit (CAM-ICU) [20], evidence of nosocomial infections including antibiotic utilization and positive microbial cultures. On discharge from ICU and during the patient’s remaining hospitalization the same data were collected with modifications for the ward environment (Confusion Assessment Method [21], highest level of mobility of that day categorized as bed, up to chair, walking with assistance or walking independently). Frailty was again assessed at hsopital discharge using the CFS or FI. Patients were urged to return for an in-person follow-up at 6 months where we again determined their frailty status using the CFS and outcomes including mortality and morbidity, indices of healthcare utilization (including hospital readmission and emergency room visits), need for institutionalization and quality of life (Euro-QoL 5D-5L [22]). All the data were collected electronically using a REDCAP database hosted at Queens University [23].

### Statistical analysis

In order to be able to conduct a large multicenter study, we defined feasibility as an enrollment rate greater than 1 per week and a consent rate of 70%. To be able to evaluate feasibility, we aimed for a convenience sample of 50 patients for the pilot. For reporting of data, continuous variables are reported as means and standard deviations (SD), except for skewed variables which are reported as means, quartiles and ranges. Categorical variables are reported as counts and percentages.

### Ethics

Research Ethics Board (REB) approval was obtained from Queens University Health Sciences Research Ethics Board. If the patient was able to consent, consent and data were obtained from the patient. If the patient was unable to consent, consent was obtained from the substitute decision-maker. When the patient regained capacity, consent for ongoing participation was obtained from them for further data collection.

## Results

**Fig. 1:**
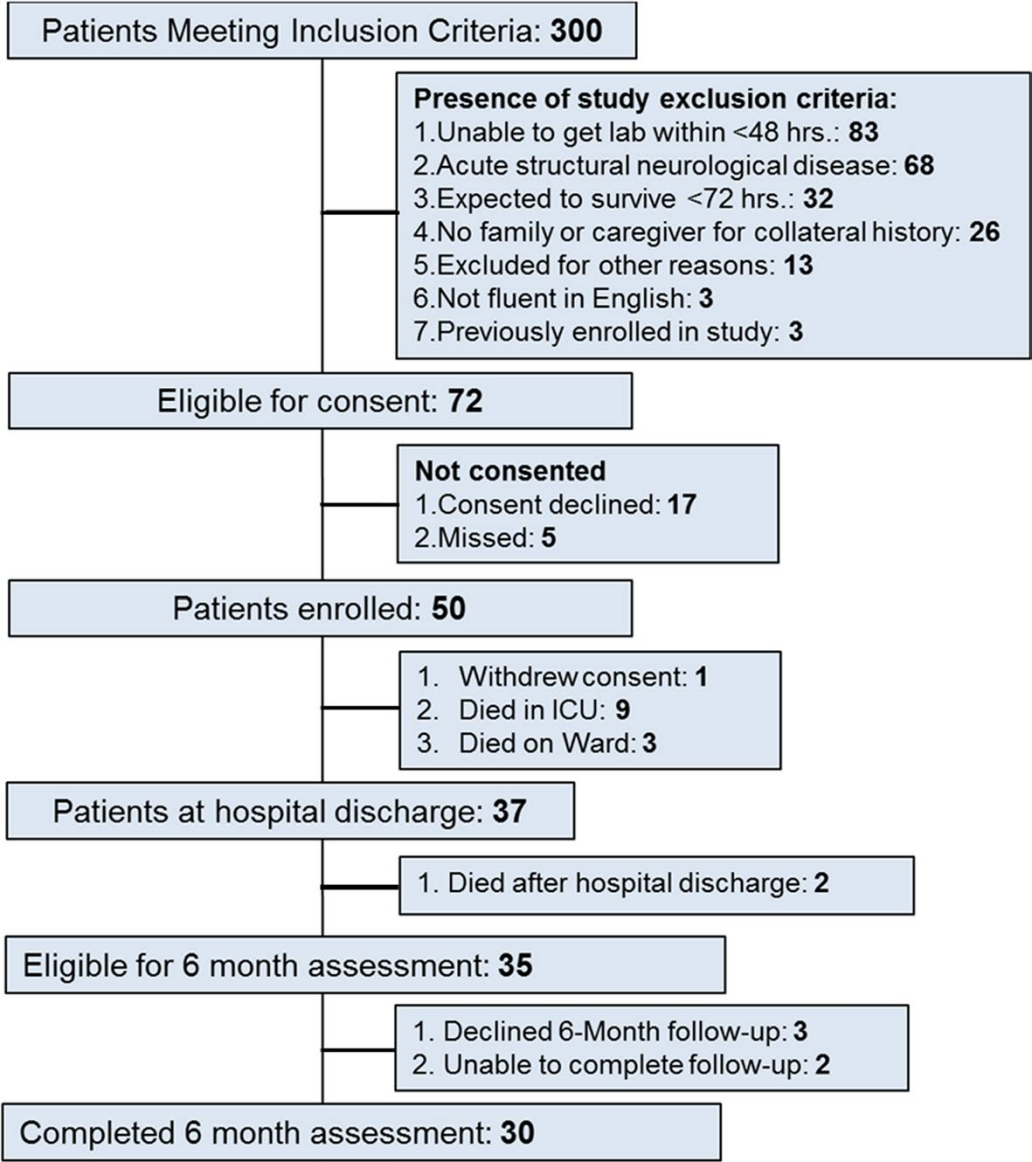
Consort diagram

Fifty patients were enrolled in the study; 1 withdrew consent and 12 people died leaving 37 patients at hospital discharge, as outlined in Fig. 1. Of these 35 patients survived to the 6-month follow-up visit and 30 were available for assessment. The characteristics of the enrolled cohort are found in Table 1. The mean (SD) age was 68.7 ± 7.9, with the majority being male and a medical basis for their admission.

**Table 1:** Baseline characteristics

	N = 49
Age: mean ± SD	68.7 ± 7.9
Sex: female—*n* (%)	18 (36.7%)
Admission type—*n* (%)	
Medical	37 (75.5%)
Surgical (emergency)	9 (18.4%)
Surgical (elective)	3 (6.1%)
Primary diagnosis	
Respiratory	19 (38.8%)
Sepsis	9 (18.4%)
Gastrointestinal	9 (18.4%)
Neurologic	4 (8.2%)
Misc	8 (16.3%)
APACHE II: mean ± SD	22.3 ± 6.2
Charlson Comorbidity Index: mean ± SD	1.8 ± 1.5
Short IQCODE: mean ± SD	3.1 ± 0.3
Infection within 2 days of ICU admit: *n* (%)	36 (73.5%)
Antibiotics within 2 days of ICU admit: *n* (%)	44 (89.8%)
Frailty Index ≥ 0.2	23 (46.9%)
Frailty Index: mean ± SD	0.2 ± 0.1
Clinical Frailty Score > 4: *n* (%)	17 (34.7%)
Frailty Score: mean ± SD	3.8 ± 1.8

*SD* standard deviation, *APACHE* acute physiology and chronic health evaluation, *IQCODE* informant questionnaire on cognitive decline in the elderly

For the primary outcome of feasibility, the consent rate was 74% with an enrollment rate of 1 patient/per week. Processes of care were readily captured during the ICU and during the patient’s ward stay with the exception of nutrition. During the ‘patients’ ward stay we were unable to capture nutrition data in spite of multiple attempts including direct observation and calorie counts conducted by dietary staff. Barriers included variability in the timing of meals and collection of meal leftovers, provision of food by family members, low rate of calorie count collection and concern over the accuracy of calorie counts. Of the patients alive at 6 months (35), 20 (57%) were able to return for in-person follow-up.

**Fig. 2:**
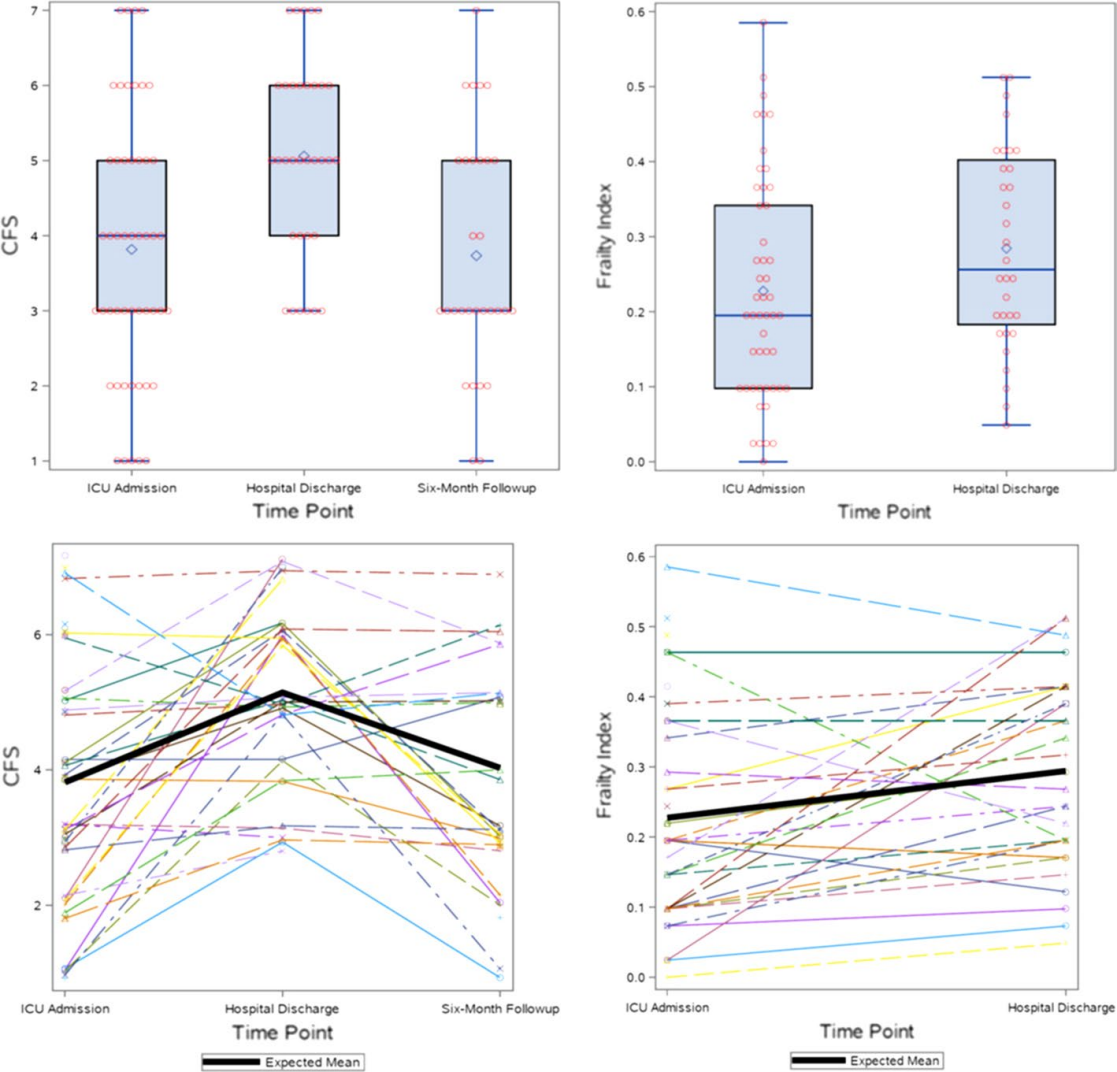
Frailty over time

The classification of frailty varied by the method used and when the frailty assessment was conducted (Table 2). Frailty was present on admission when using the CFS (CFS ≥ 5) in 17/49 (36%) and in 23/49 (47%) using the FI. On discharge from hospital 22/33 (67%) were categorized as frail using the CFS in contrast to 21/33 (63%) using the FI. Discharge assessments were missed in 4 patients. Individual courses of frailty are plotted in Fig. 2. At 6 months, 11 had a CFS ≥ 5. The CFS rose over the course of hospital but tended to return to baseline at the 6-month time-point; of the 23 patients who had a baseline CFS < 5, only 5 (22%) had a CFS ≥ 5 at 6 months.

**Table 2:** Frailty characterization on ICU admission and hospital discharge

	CFS not frail (< 5) *n* (%) (mean ± SD)	CFS frail (≥ 5) *n* (%) (mean ± SD)	FI not frail (< 0.2) *n* (%) (mean + SD)	FI not frail (≥ 0.2) *n* (%) (mean + SD)
Admission (*n* = 49)	32 (65%) (2.8 ± 1.0)	17 (36%) (5.8 ± 0.8)	26 (53%) (0.1 ± 0.1)	23 (47%) (0.4 ± 0.1)
Discharge (*n* = 33)^a^	11 (33%) (3.2 ± 0.9)	22 (67%) (5.7 ± 0.8)	12 (36%) (0.1 ± 0.05)	21 (63%) (0.4 ± 0.1)
Mortality	4 (13%)	8 (47%)	1 (4%)	11 (48%)

*CFS* clinical frailty Scale, *FI* frailty index

^a^12 patients died during the hospitalization and frailty assessments missed in 4 patients

Processes of care are outlined in Table 3. Outcomes classified by FI are outlined in Table 4 and outcomes classified by CFS are reported in Table 5. Overall, whether frailty was assessed by FI or CFS, the in-hospital outcomes and follow-up outcomes were worse in those who were classified as frail. In addition, the level of frailty appeared to remain static in survivors with pre-existing frailty but seemed to progress in non-frail survivors over the course of hospitalization and by the 6-month follow-up. Outcomes categorized by discharge frailty assessment are presented in Additional file 2: Appendix 2, Table S1. Patients identified as frail on discharge had fewer 28-day ICU-free days, were less likely to be discharged home and more like to be discharged to another facility including long-term care or a rehabilitation facility. The differences in 6-month mortality or quality of life between those that were or not frail on hospital discharge were not statistically significant.

**Table 3:** Care processes and adverse events during ICU and hospital stay

	FI ≥ 0.2 (*n* = 23)	FI < 0.2 (*n* = 26)	*p* value^1^	CFS ≥ 5 (*n* = 17)	CFS < 5 (*n* = 32)	*p* value^1^	Total (*n* = 49)
Nutrition evaluable days: mean ± SD	10.2 ± 9.8	8.1 ± 8.9	0.4	12.2 ± 9.2	7.4 ± 9.0	0.09	9.1 ± 9.3
Received TPN: *n* (%)	2 (8.7)	4 (15.4)	0.7	2 (11.8)	4 (12.5)	1.0	6 (12.2)
EN caloric adequacy (%): mean ± SD	44.8 ± 34.5	50.7 ± 35.0	0.6	62.4 ± 22.8	39.8 ± 37.5	0.03	47.8 ± 34.5
EN protein adequacy (%): mean ± SD	44.3 ± 34.3	50.4 ± 35.6	0.6	62.2 ± 24.9	39.3 ± 37.0	0.03	47.4 ± 34.7
Mobility at ICU discharge^2^: mean ± SD	3.1 ± 3.5	5.3 ± 3.9	0.04	3.1 ± 4.0	5.0 ± 3.6	0.1	4.3 ± 3.8
Proportion ward days patient out of bed at least once: median (IQR) or mean ± SD	0.3 (0.2–0.8)	0.7 (0.2–1.0)	0.27	0.7 ± 0.4	0.5 ± 0.4	0.5	0.5 (0.2–1.0)
Maximum level of activity over ward stay: missing	10 (43.5%)	3 (11.5%)		8 (47.1%)	5 (15.6%)		13 (26.5%)
Ambulatory	11 (47.8%)	20 (76.9%)		7 (41.2%)	24 (75.0%)		31 (63.3%)
Up to a chair	0 (0.0%)	3 (11.5%)		1 (5.9%)	2 (6.3%)		3 (6.1%)
Bed	2 (8.7%)	0 (0.0%)		1 (5.9%)	1 (3.1%)		2 (4.1%)
Proportion ICU days with CAM assessed: median (IQR)	0.8 (0.7–1.0)	0.9 (0.6–1.0)	0.6	0.9 [0.6–0.9)	0.9 [0.7–1.0)	0.5	0.9 (0.7–1.0)
Proportion ICU days with CAM + ve score: median (IQR)	0.2 (0.0–0.5)	0.1 (0.0–0.4)	0.2	0.2 (0.0–0.5)	0.2 (0.0–0.4)	0.4	0.2 (0.0–0.5)
Proportion ward days with CAM assessed: median (IQR)	1.0 (1.0–1.0)	1.0 (1.0–1.0)	0.3	1.0 (1.0–1.0)	1.0 (0.96–1.0)	0.04	1.0 (1.0–1.0)
Proportion ward days with CAM + ve score: median (IQR)	0.0 (0.0–0.0)	0.0 (0.0–0.0)	1.0	0.0 [0.0–0.0)	0.0 (0.0–0.0)	1.0	0.0 [0.00. 0 (0.0–0.0)
Number new antibiotics ICU day 2–28: median (IQR)	1.0 (0.0–0.0)	1.0 (0.0–3.0)	0.867	1.0 (0.0–2.0)	0.0 (0.0–1.0)	0.361	1.0 (0.0–2.0)
Number of positive cultures ICU day 2–28: median (IQR)	1.0 (0.0–2.0)	0.0 (0.0–2.0)	0.544	1.0 (0.0–2.0)	1.0 (0.0–3.0)	0.614	0.0 (0.0–2.0)

*FI* Frailty Index, *CFS* Clinical Frailty Score, *SD* Standard Deviation, *TPN* Total Parenteral Nutrition, *EN* Enteral Nutrition, *IQR* Interquartile Range, *ICU* Intensive Care Unit,

^1^*p*-values are Fisher's exact test for categorical variables and t-test or Wilcoxon rank-sum test for continuous variables

^2^As measured by the ICU Mobility Scale (ranges from 0 = bed bound to 10 = able to walk independently)

**Table 4:** Outcomes by admission frailty index

	Admission frailty index ≥ 0.2 (*n* = 23)	Admission frailty index < 0.2 (*n* = 26)	Total (*n* = 49)	*p* value*
FI on ICU admission: mean ± SD	0.4 ± 0.1	0.1 ± 0.1	0.2 ± 0.1	< 0.001
FI on hospital discharge: mean ± SD	0.3 ± 0.1	0.2 ± 0.1	0.3 ± 0.1	0.034
Change in FI from admission to discharge: mean ± SD	− 0.0 ± 0.1	0.1 ± 0.1	0.1 ± 0.1	0.003
CFS on ICU admission: mean ± SD	5.1 ± 1.3	2.7 ± 1.3	3.8 ± 1.8	< 0.001
CFS at 6-month follow-up: mean ± SD	4.6 ± 1.3	3.3 ± 1.6	3.7 ± 1.6	0.033
FI on hospital discharge				< 0.001
< 0.2	1 (4.3%)	11 (42.3%)	12 (24.5%)	
≥ 0.2	10 (43.5%)	10 (38.5%)	20 (40.8%)	
Unknown	1 (4.3%)	4 (15.4%)	5 (10.2%)	
Length of ICU stay (days): median (IQR)	12.0 (6.0–20.0)	7.5 (5.0–14.0)	9.0 (5.0–16.0)	0.366
Length of hospital stay (days): median (IQR)	23.0 (13.0–38.0)	25.5 (13.0–51.0)	25.0 (13.0–39.0)	0.912
28 Day ICU-free days: median (IQR)	0.0 (0.0–19.0 0)	20.5 (13.0–23.0)	16.0 (0.0–22.0)	0.005
Hospital mortality: *n* (%)	11 (47.8%)	1 (3.8%)	12 (24.5%)	< 0.001
Hospital discharge destination				0.437
Home	8 (34.8%)	18 (69.2%)	26 (53.0%)	
Rehabilitation Center	2 (8.7%)	4 (15.4%)	6 (12.2%)	
Long-term care facility	1 (4.3%)	2 (7.7%)	3 (6.1%)	
Other acute care hospital	1 (4.3%)	1 (3.8%)	2 (4.1%)	
6-month mortality: *n* (%)	12 (52.2%)	2 (7.7%)	14 (28.6%)	0.001
6-month Quality of Life (EQ-5D-5L Index): mean ± SD	0.6 ± 0.2	0.8 ± 0.2	0.7 ± 0.2	0.047

*FI* frailty index, *CFS* clinical frailty scale, *SD* standard deviation, *ICU* intensive care unit, *IQR* interquartile range

^*^p-values are Fisher’s exact test for categorical variables and t-test or Wilcoxon rank-sum test for continuous variables

**Table 5:** Outcomes by admission clinical frailty scale

	Admission Frailty Score ≥ 5 (*n* = 17)	Admission Frailty Score < 5 (*n* = 32)	Total (*n* = 49)	*p* value*
CFS on ICU admission: mean ± SD	5.8 ± 0.8	2.8 ± 1.0	3.8 ± 1.8	< 0.001
CFS on hospital discharge: mean ± SD	5.1 ± 1.6	4.8 ± 1.4	4.9 ± 1.4	0.631
Change in CFS from admission to discharge: mean ± SD	− 0.5 ± 1.5	2.2 ± 1.7	1.4 ± 2.0	< 0.001
CFS at 6-month follow-up: mean ± SD	5.3 ± 1.3	3.3 ± 1.4	3.7 ± 1.6	0.002
FI on ICU admission: mean ± SD	0.4 ± 0.1	0.1 ± 0.1	0.2 ± 0.1	< 0.001
CFS 5 or above at hospital discharge: *n* (%)				0.014
< 5	1 (5.9%)	10 (31.3%)	11 (22.4%)	
≥ 5	8 (47.1%)	14 (43.8%)	22 (44.9%)	
Unknown	0 (0.0%)	4 (12.5%)	4 (8.2%)	
Length of ICU stay (days): median (IQR)	13.0 (9.0–20.0)	6.0 (4.0–12.5)	9.0 (5.0–16.0)	0.030
Length of hospital stay (days): median (IQR)	26.0 (17.0–38.0)	24.0 (12.5–43.0)	25.0 (13.0–39.0)	0.644
28-day ICU-free days: mean ± SD	7.2 ± 9.0	15.0 ± 10.3	12.3 ± 10.4	0.012
Hospital mortality: *n* (%)	8 (47.1%)	4 (12.5%)	12 (24.5%)	0.013
Discharge location: *n* (%)				0.679
Home	5 (29.4%)	24 (65.7%)	26 (53.0%)	
Rehabilitation center	2 (11.8%)	4 (12.5%)	6 (12.2%)	
Long-term care facility	1 (5.9%)	2 (6.3%)	3 (6.1%)	
Other acute care hospital	1 (5.9%)	1 (3.1%)	2 (4.1%)	
6-month mortality: *n* (%)	9 (52.9%)	5 (15.6%)	14 (28.6%)	0.009
Six-month Quality of Life (EQ-5D-5L Index): mean ± SD	0.5 ± 0.2	0.8 ± 0.2	0.7 ± 0.2	0.005

*CFS* clinical frailty scale, *FI* frailty index, *SD* standard deviation, *ICU* intensive care unit, *IQR* interquartile range

^*^p-values are Fisher's exact test for categorical variables and t-test or Wilcoxon rank-sum test for continuous variables

## Discussion

In this single-center pilot study, we met the a priori enrollment criteria set as necessary to feasibly conduct a multicenter study and were able to develop the data collection tools required for its’ conduct. We also demonstrated that frailty identification varied with the tool used and was dynamic through the ICU and hospital course. Further, the identification of frailty, irrespective of the method used was associated with worse outcomes. Due to the small sample size of this pilot study and its single-center study design, these results are hypothesis generating only but are corroborated by the findings of other studies [4, 9, 10, 24, 25]. This study is novel in its measurement of frailty at different times points along with the process of care received and potentially adverse events for which frail patients may be at higher risk for. Although the data generated are preliminary and need to be verified in a larger multicenter study, they begin to fill some of the knowledge gaps for the interaction between critical illness and frailty.

In this pilot study, enrollment was 1 patient per week. Recognizing that a substantial number of patients of potentially eligible patients were not enrolled and in discussion with the steering committee, the inclusion and exclusion criteria were substantially revised for the multicenter study. In particular, the exclusion of patients with neurological disease was removed. In addition, the timelines for study entry were significantly lengthened to 5 days to reduce potential participants lost on weekends and holidays. In addition, recognizing that a significant number of patients were not able to return for in-person follow-up, for the multicenter study, both in-person and telephone follow-up will be allowed. These changes will facilitate recruitment and increase the generalizability of study findings. For planning purposes, we estimated that enrollment in the multicenter study will be 1.25 patients/week.

In the multicenter study, we aim to further describe the outcomes associated with the method and timing of frailty ascertainment in addition to the impact of processes of care on frailty outcomes. The analyses to answer our research questions will involve the construction of multi-variate models and we will need to have an adequate number of patients with the characteristics of interest including frailty on ICU admission and hospital discharge. Frailty at hospital discharge can be categorized as that persisting from ICU admission, unchanged, worsened or improved or newly developed, persistent or transitory as a consequence of the critical illness. A priori, the following co-variates may be important for the development or progression of frailty: admission variables (age, sex, body mass index, number of co-morbidities, presence of disability, severity of illness,, presence of cognitive impairment, admission FI, admission CFS), process of care variables (duration of life-support interventions, nutrition received as percentage of recommended amounts, mobility levels, sedation levels) and adverse events (nosocomial infections, occurrence and duration of delirium). For multi-variable models, it is desirable to have at least 10 events for every co-variate [26].

In this pilot study, approximately 40% of patients who were not frail on ICU admission (both by FI and CFS) survived, but also were found to be frail on hospital discharge. Further hospital mortality in those who were frail on ICU admission (both by FI and CFS) was approximately 47% which is higher than in some larger studies. In a meta-analysis of ICU studies, patients with frailty were reported to have a hospital mortality of 30% [4].

Given the uncertainty, for the multicenter study, we will utilize an event driven sample size where we will recruit patients until we have recruited at least 120 frail survivors and at least 150 newly developed frail survivors at hospital discharge. Assuming a mortality rate of between 30 and 45% in enrolled frail patients, between 170 and 220 patients with frailty would need to be enrolled to have 120 frail survivors. To have at least 150 patients with newly developed frailty at hospital discharge, we would need to enroll approximately 375 non-frail patients. In our pilot study, 36% of patients were frail based on the CFS and 49% were frail based on the FI. With a sample size of 700 patients, we expect to have 250–340 frail patients, at least 130 frail survivors to hospital discharge and at least 180 patients with newly developed frailty.

Strengths of this study include the measurement of frailty using different methods at different time points during critical illness and the collection of data outside the ICU. Limitations of this study included its single-center design, significant loss of patients to follow-up and that it was not powered for detailed analyses. All the results observed are hypothesis generating and will need to be confirmed in the larger multicenter study that will be adequately powered to control for important co-variates.

In conclusion, a large multicenter study studying the measurement of frailty in critically ill patients with different ascertainment tools at both ICU admission and hospital discharge was determined to be feasible and is currently in progress. The processes and data collection tools developed for the pilot study are being used in the multicenter study. The results of this pilot study have also been used to inform the sample size estimates for the multicenter study. This pilot study provides is an important step forward in elucidating the interaction between frailty and critical illness. It is only by better understanding frailty in critically ill patients including how to ascertain it and the impact of processes of care on its outcomes that we can design interventions that will improve outcomes.

## Supplementary Information

**Additional file 1:** **Appendix 1.** Frailty Index Items.

**Additional file 2:** **Appendix 2. Table S1.** Outcomes by Discharge Frailty Index.

## Data Availability

The data generated during the current study are not publicly available pending the conduct of the multicenter study but are available from the corresponding author on reasonable request.

## Abbreviations

ICU: Intensive care unit
CFS: Clinical frailty scale
FI: Frailty index
CPR: Cardio-pulmonary resuscitation
APACHE: Acute physiology and chronic health evaluation
IQCODE: Informant questionnaire on cognitive decline in the elderly
CAM: Confusion assessment method
SD: Standard deviation
