# Correction: The frailty, outcomes, recovery and care steps of critically ill patients (FORECAST) study: pilot study results

**DOI:** 10.1186/s40635-022-00487-y

**Published:** 2023-01-05

**Authors:** John Muscedere, Sean M. Bagshaw, Gordon Boyd, Stephanie Sibley, Patrick Norman, Andrew Day, Miranda Hunt, Darryl Rolfson

**Affiliations:** 1 Department of Critical Care Medicine, Queens University, Kingston Health Sciences Center, 76 Stuart Street, Kingston, ON K7L 2V7 Canada; 2 Department of Critical Care Medicine, University of Alberta, Edmonton, Canada; 3 Kingston Health Sciences Center, Kingston, ON Canada; 4 University of Alberta, Edmonton, Canada

**Correction: Intensive Care Medicine Experimental (2022) 10:23** **https://doi.org/10.1186/s40635-022-00446-7**

Following publication of the original article [1], it came to the authors' attention that the first and last name of the 5th author, Patrick Norman, had been erroneously swapped (to Norman Patrick). The name has since been corrected in the published article and the corrected name may be found in this erratum.
