# Supplementary material for: The frailty, outcomes, recovery and care steps of critically ill patients (FORECAST) study: pilot study results
Source: Intensive Care Med Exp. 2022 Jun 10;10:23. doi: 10.1186/s40635-022-00446-7 (PMC9184687; doi:10.1186/s40635-022-00446-7)
Supplement: Supplementary file 1 — Additional file 1: Appendix 1. Frailty Index Items. [file 40635_2022_446_MOESM1_ESM.pdf]

Appendix 1 - Frailty Index Items[1]

#	**Items contributed to the FI**
1	Overall health of the patient?
2	Do you think the patient was depressed?
3	Do you think the patient worries a lot or is anxious?
4	Do you think the patient felt exhausted or tired all the time?
5	Did the patient have sleep problems?
6	Did the patient have problems with memory or thinking?
7	Did the patient have any problems speaking to make him/herself understood?
8	Did the patient have difficulty hearing?
9	Did the patient have problems with eyesight? (even when wearing glasses)
10	Did the patient report having problems with balance?
11	Did the patient complain of feeling dizzy or lightheaded?
12	Did the patient need assistance of a person or aid to prevent falling?
13	Did the patient hold on to furniture to keep from failing?
14	Was the patient able to walk alone?
15	Was the patient able to get out of a bed or chair alone?
16	Did the patient have problems with bowel control?
17	Did the patient have problems with bladder control?
18	Did the patient experience any unplanned weight loss in the last 6 months?
19	What was the patient’s food intake in the week prior to ICU admission?
20	Was the patient able to carry out some day to day tasks?
21	Feed himself/herself?
22	Take a bath or shower?
23	Dress himself/herself?
24	Drive?
25	Look after his/her own medications?
26	Do day-to-day shopping?
27	Do day-to-day household cleaning?
28	Cook well enough to maintain his/her nutrition?
29	Look after his/her own banking and financial affairs?
30	Myocardial infarct
31	Congestive heart failure
32	Peripheral vascular disease
33	Cerebrovascular disease +/- hemiplegia
34	Dementia
35	Chronic pulmonary disease
36	Connective tissue disease
37	Peptic ulcer disease
38	Any liver disease
39	Diabetes
40	Diabetes with end organ damage
41	Moderate or several renal diseases
42	Any tumor

1. Heyland DK, Garland A, Bagshaw SM, Cook D, Rockwood K, Stelfox HT, Dodek P, Fowler RA, Turgeon AF, Burns K, Muscedere J, Kutsogiannis J, Albert M, Mehta S, Jiang X, Day AG, (2015) Recovery after critical illness in patients aged 80 years or older: a multi-center prospective observational cohort study. Intensive Care Med 41: 1911-1920
