# Supplementary material for: The frailty, outcomes, recovery and care steps of critically ill patients (FORECAST) study: pilot study results
Source: Intensive Care Med Exp. 2022 Jun 10;10:23. doi: 10.1186/s40635-022-00446-7 (PMC9184687; doi:10.1186/s40635-022-00446-7)
Supplement: Supplementary file 2 — Additional file 2: Appendix 2. Table S1. Outcomes by Discharge Frailty Index. [file 40635_2022_446_MOESM2_ESM.docx]

**Table S1 - Outcomes by Discharge Frailty Index**

|  | **Discharge**  **FI ≥ 0.2 (n=20)** | **Discharge**  **FI < 0.2 (n=12)** | **Total (n=32)** | **P-value*** | **Discharge CFS ≥ 5 (n=23)** | **Discharge** **CFS < 5 (n=10)** | **Total (n=33)** | **P-value*** |
| --- | --- | --- | --- | --- | --- | --- | --- | --- |
| **FI on ICU Admission:** *mean ± SD* | 0.2 ±0.1 | 0.1 ±0.1 | 0.2 ±0.1 | **0.046** | **--** | **--** | **--** | **--** |
| **FI on Hospital Discharge:** *mean ± SD* | 0.4 ±0.1 | 0.1 ±0.1 | 0.3 ±0.1 | **<.001** | **--** | **--** | **--** | **--** |
| **CFS on ICU Admission:** *mean ± SD* | **--** | **--** | **--** | **--** | 3.9 ±1.6 | 2.8 ±1.7 | 3.5 ±1.7 | 0.086 |
| **CFS on Hospital Discharge:** *mean ± SD* | **--** | **--** | **--** | **--** | 5.4 ±1.3 | 4.4 ±1.1 | 5.0 ±1.3 | **0.047** |
| **CFS at 6-month follow-up:** *mean ± SD* | **--** | **--** | **--** | **--** | 4.4±1.5 | 3.1±1.6 | 3.8±1.6 | **0.035** |
| **Length of ICU stay (days):** *median* *(IQR)* | 8.5  (5.5, 22.5) | 6.5  (4.5, 14.5) | 8.0  (5.0, 17.0) | 0.519 | 8.5  (5.5, 22.5) | 6.5  (4.5, 14.5) | 8.0  (5.0, 17.0) | 0.519 |
| **Length of hospital stay (days):** *median (IQR)* | 32.0  (13.0, 70.0) | 21.0  (10.0, 31.5) | 25.5  (13.0, 43.0) | 0.129 | 32.0  (13.0, 70.0) | 21.0  (10.0, 31.5) | 25.5  (13.0, 43.0) | 0.129 |
| **28 Day ICU-free days:** *median*  *(IQR)* | 19.5  (5.5, 22.5) | 21.5  (13.5, 23.5) | 20.0  (11.0, 23.0) | 0.44 | 16.0  (5.0, 22.0) | 23.0  (21.0, 25.0) | 20.0  (10.0, 23.0) | **0.039** |
| **Discharge Location** |  |  |  | 0.159 |  |  |  | **0.003** |
| Home | 12 (60.0%) | 12 (100%) | 24 (75.0%) |  | 14 (60.9%) | 10 (100.0%) | 24 (73.2%) |  |
| Rehabilitation Centre | 5 (25.0%) | 0 (0.0%) | 5 (15.6%) |  | 5 (21.7%) | 0 (0.0%) | 5 (15.2%) |  |
| Complex continuing care hospital | 2 (10.0%) | 0 (0.0%) | 2 (6.3%) |  | 3 (13.0%) | 0 (0.0%) | 3 (9.1%) |  |
| Other acute care hospital | 1 (5.0%) | 0 (0.0%) | 1 (3.1%) |  | 1 (4.3%) | 0 (0.0%) | 1 (3.0%) |  |
| **6-month mortality:** *n (%)* | 2 (10.0%) | 0 (0.0%) | 2 (6.3%) | 0.516 | 2 (8.7%) | 0 (0.0%) | 2 (6.1%) | 1 |
| **6 Month QoL (EQ-5d-5L Index):**  *mean ± SD* | 0.7 ± 0.3 | 0.8 ± 0.2 | 0.7 ± 0.2 | 0.231 | 0.7 ± 0.2 | 0.8 ± 0.2 | 0.7 ± 0.2 | 0.214 |

* p - values are Fisher's exact test for categorical variables and t-test or Wilcoxon Rank-Sum test for continuous variables.

Abbreviations: FI = Frailty Index, CFS = Clinical Frailty Scale, SD = Standard Deviation, ICU = Intensive Care Unit, IQR = Interquartile Range, QoL = Quality of Life
